# Cannabis use and its psychosocial correlates among school-going adolescents in Sierra Leone

**DOI:** 10.1186/s12889-024-18491-0

**Published:** 2024-04-08

**Authors:** Augustus Osborne, Peter Bai James, Comfort Z. Olorunsaiye, Camilla Bangura, Aiah Lebbie

**Affiliations:** 1 Department of Biological Sciences, School of Basic Sciences, Njala University, PMB, Freetown, Sierra Leone; 2 National Centre for Naturopathic Medicine, Faculty of Health, Southern Cross University, Lismore, Australia; 3 Faculty of Pharmaceutical Sciences, College of Medicine and Allied Health Sciences, University of Sierra Leone, Freetown, Sierra Leone; 4 Department of Public Health, Arcadia University, 450 S Easton Road, Glenside, PA 19038 USA

**Keywords:** School-going adolescents, Cannabis use, Prevalence, Sierra Leone

## Abstract

**Background:**

In Sierra Leone, adolescents are increasingly engaging in risky activities, including cannabis use, which can lead to substance abuse, poor academic performance, and psychotic symptoms. This study aims to investigate the prevalence and associated factors of cannabis use among school-going adolescents in the country.

**Method:**

Data for the study was sourced from the 2017 Sierra Leone Global School-based Student Health Survey (GSHS), a nationally representative survey conducted among adolescents aged 10-19 years using a multistage sampling methodology. A weighted sample of 1,467 adolescents in Sierra Leone was included in the study. The study utilised bivariate and multivariable logistic regression analysis to identify factors linked to cannabis use, presenting results using adjusted odds ratios and 95% confidence intervals.

**Results:**

The prevalence of cannabis use was 5.1% [3.0,8.6] among school-going adolescents in Sierra Leone. School-going adolescents who attempted suicide [aOR = 6.34, 95% CI = 1.71–23.45], used amphetamine [aOR = 15.84, 95% CI = 7.94–31.62] and were involved in sexual risk behaviour [aOR = 5.56, 95% CI = 2.18–14.20] were more likely to be associated with cannabis use.

**Conclusion:**

In Sierra Leone, a small but non-trivial minority of students use cannabis. Ever-used amphetamines or methamphetamines, suicidal attempts, and sexual risk behaviour were the factors associated with cannabis use in Sierra Leone. The development of school-based health intervention programmes is crucial to address the risk factors associated with cannabis use among school-going adolescents.

**Supplementary Information:**

The online version contains supplementary material available at 10.1186/s12889-024-18491-0.

## Introduction

Cannabis is the most often used illegal drug worldwide [1]. An estimated 192 million people aged 15–64 was using it in 2018 [1]. Among adolescents aged 15–19 years, the global annual prevalence of cannabis use was 13.8 per cent in 2018, which translates to about 45 million adolescents [1]. In Africa, cannabis remains the most prevalent illicit substance, with the highest rates of use reported in West and Central Africa [1]. Some adverse effects of cannabis use include impaired cognitive function, increased risk of psychotic disorders, reduced academic achievement, and involvement in other risky behaviours [2].

Cannabis use among adolescents varies between countries in Africa. A study by Bangura et al. [3] reported that the prevalence of cannabis use was 2.2% among school-going pupils in Bo, southern Sierra Leone. According to a study by Asante [4] based on the 2012 Ghanaian Global School-based Student Health Survey, the past-month prevalence of cannabis use among school-going adolescents aged 11–19 years was 5.3%. In another study in Zambia by Siziya et al., [5] the overall prevalence of self-reported ever-used cannabis was 37.2%. In Port Harcourt, Nigeria [6], 26% of secondary school students use cannabis, while in Ilorin, Nigeria, 3.4% use it [7]. In Zimbabwe [8, 9], 3.4% and 6.2% of rural and urban secondary school students have used cannabis, while in KwaZulu-Natal, South Africa [10], 16.9% of high school students have. These variations may be due to differences in definitions of cannabis use prevalence in Africa.

Cannabis use among adolescents is linked to factors such as male gender [5, 11, 12], older age [11, 13], mental health issues [5, 12, 14–17], alcohol use [5], and current smoking [12, 18]. School social dynamics, such as bullying [5, 19], physical fighting [19], school truancy [4, 18–20], lack of peer support [18], and having many friends [4, 21, 22], also contribute to cannabis use. Parental characteristics, such as substance use [14, 23], lack of parental support [18], and maternal demandingness [12], also influence cannabis use among school-going adolescents. Therefore, parents must provide support and monitoring to help adolescents navigate the complexities of cannabis use and its impact on their health.

In Sierra Leone, the use of cannabis is prohibited, adhering to the Single Convention on Narcotic Drugs of 1961 [24]. Cannabis cultivation, however, remains widespread throughout Sierra Leone, and various forms of cannabis use are prevalent throughout the country. Currently, Sierra Leone lacks legislation or interventions that specifically address the issue of cannabis use among adolescents [25]. However, the absence of targeted programmes for school-going adolescents does not imply a complete lack of effort to tackle the issue. Non-governmental Organisations and community organisations provide harm reduction programmes that indirectly help school-going adolescents who may be facing substance misuse issues, including cannabis [25].

Cannabis use among adolescents in Sierra Leone is under-researched, with one study [3] done among school-going pupils in Bo, southern Sierra Leone. However, this study has limitations due to demographic transitions and urbanisation, and its findings may not represent the entire country. We used the 2017 Global School-Based Health Survey data to estimate the prevalence and sociodemographic correlates of cannabis use among school-going adolescents in Sierra Leone. This study will contribute to the literature on illicit drug use in developing nations and inform public health policymakers and program planners on how to prevent or reduce adolescent drug use.

## Materials and methods

### Sample and procedure

The 2017 Global School Health Survey in Sierra Leone provided a cross-sectional dataset we could utilise [26]. The study employed a two-stage cluster sampling methodology to obtain a representative dataset encompassing all students aged 10-19 years in grades JSS 2, JSS 3, SSS 2, and SSS 3 in Sierra Leone. The first step includes picking schools with a probability proportional to students' enrolment. The second stage involves randomly selecting classes for all students, ensuring an equal chance of selection. The response rate for Sierra Leone's GSHS schools was 94%, and the student response rate was 87% [26]. The Sierra Leone Global School-based Student Health Survey (GSHS) involved a total of 2,798 student participants. The detailed GSHS description can be retrieved via https://extranet.who.int/ncdsmicrodata/index.php/catalog/772/study-description (Accessed August 20, 2023). A weighted sample of 1,467 school-going adolescents aged 10-19 years was included in our final analysis. Our study aligns with STROBE guidelines for reporting observational studies (See Supplementary file 1).

### Outcome variable

The study’s dependent variable was cannabis use. The original question was, “During your life, how many times have you used marijuana” Response options ranged from 1, representing zero times, through 5, representing 20 or more times. We recoded 1=0 and 2-5=1 for the analysis. The replies were classified based on the previous research in Ghana [4].

### Explanatory variables

The explanatory variables employed in the analysis were selected because of their availability in the GSHS dataset and historical connections with the dependent variables. Age, gender, grade, truancy, alcohol use, suicidal ideation, suicidal plan, suicide attempts, loneliness, anxiety, being bullied, peer support, close friends, and parental support were the explanatory variables examined. Our research variables have been widely utilised and verified in prior research [4]. (Table 1) displays a thorough explanation of the variables and the recoded replies.

**Table 1:** Cannabis use and its psychosocial correlates among school-going adolescents in Sierra Leone

Variable	Survey question	Original response options	Recoded
Outcome Variable (Cannabis use)			
Cannabis use	During your life, how many times have you used marijuana	1 = 0 times 2=1 or 2 times 3= 3 to 9 times 4=10 to 19 times 5 = 20 or more times	1 = 0 and 2–5 = 1
Independent Variables			
Age	How old are you?	1=11 years old or younger 2= 12 years old 3= 13 years old 4= 14 years old 5= 15 years old 6= 16 years old 7= 17 years old 8= 18 years old or older	≤14 years= 1 and ≥15 years=2
Gender	What is your sex?	1 = male; 2 = female	1 = male; 2 = female
Grade	In what grade are you?	1= Jnr Sec (JSS) 2 2= Jnr Sec (JSS) 3 3= Snr Sec (SSS) 1 4= Snr Sec (SSS) 2 5= Snr Sec (SSS) 3	1=1-2 and 2=3-5
Amphetamine use	During your life, how many times have you used amphetamines or methamphetamine	1 = 0 times 2=1 or 2 times 3= 3 to 9 times 4=10 to 19 times 5 = 20 or more times	1 = 1 and 2–5 = 2
Alcohol use	During the past 30 days, on how many days did you have at least one drink containing alcohol?	1 = 0 days 2= 1 or 2 days 3= 3 to 5 days 4= 6 to 9 days 5= 10 to 19 days 6= 20 to 29 days 7 = all 30 days	1 = 1 and 2–7 = 2
Suicidal Ideation	During the past 12 months, did you ever seriously consider attempting suicide?	Yes = 1; no = 2	Yes = 1 and no = 2
Suicidal plan	During the past 12 months, did you plan how you would attempt suicide?	Yes = 1; no = 2	Yes = 1 and no = 2
Suicidal attempt	During the past 12 months, how many times did you attempt suicide?	1 = 0 times 2= 1 times 3= 2 or 3 times 4= 4 or 5 times 5 = 6 times or more	1 = 1 and 2–5 = 2
Close friends	How many close friends do you have?	1 = 0 2= 1 3= 2 4 = 3 or more	1 = 1 and 2–4= 2
Loneliness	During the past 12 months, how often have you felt lonely?	1=Never 2=Rarely 3= Sometimes 4=Most of the time 5=Always	1 = 1 and 2–5 = 2
Anxiety	During the past 12 months, how often have you been so worried about something that you could not sleep at night?	1=Never 2=Rarely 3= Sometimes 4=Most of the time 5=Always	1 = 1 and 2–5 = 2
Bullied	During the past 30 days, how many days were you bullied?	1 = 0 days 2= 1 or 2 days 3= 3 to 5 days 4= 6 to 9 days 5= 10 to 19 days 6= 20 to 29 days 7 = all 30 days	1 = 1 and 2–7 = 2
Sexual risk behaviour	During your life, with how many people have you ever had sexual intercourse?	1 = never had intercourse 2= 1 person 3= 2 people 4= 3 people 5= 4 people 6= 5 people 7 = 6 or more people	1 = 1 and 2–7 = 2
School truancy	During the past 30 days, how many days did you miss classes or school without permission?	1 = 0 days 2= 1 or 2 days 3= 3 to 5 days 4= 6 to 9 days 5= 10 or more days	1 = 1 and 2–5 = 2
Peer support	During the past 30 days, how often were most of the students in your school kind and helpful?	1=Never 2=Rarely 3= Sometimes 4=Most of the time 5=Always	1 = 1 and 2–5 = 2
Parental support	During the past 30 days, how often did your parents or guardians check to see if your homework was done?	1=Never 2=Rarely 3= Sometimes 4=Most of the time 5=Always	1 = 1 and 2–5 = 2

### Statistical analyses

All statistical analyses were done using SPSS Statistics v29.0.1.0 (IBM Corp, Armonk, NY). Frequencies and percentages were first used to present the background characteristics of the school-going adolescents. We employed percentages and confidence intervals (CI) to present the prevalence and distribution of cannabis use among school-going adolescents. Prior to the regression analysis, we conducted a multicollinearity test using the variance inflation factor (VIF), and the results showed that the minimum and maximum VIFs were 1.02 and 1.47, respectively. Hence, there was no evidence of high collinearity among the variables studied. Later, a multivariable binary logistic regression analysis was adopted to examine the factors associated with cannabis use among school-going adolescents. The first model (Model I) examined the independent association between the explanatory variables and cannabis use, and the results were presented using crude odds ratio (cOR) with their respective 95% CI. All the variables that showed a statistically significant association with cannabis use were placed in Model II, the complete model. Model II results were presented using adjusted odds ratio (aOR) with 95% CI. Statistical significance was set at *p*<0.05 at the chi-square and regression analyses. All the analyses were weighted to cater to under-and-over sampling and to adjust the complex sampling methodology. All missing observations 1331 on variables of interest were dropped. We did a sensitivity analysis by running multiple imputation by chained equations (MICE) by imputing the missing data and then ran the analyses on the imputed data to compare with the results of where all missing variables of interest were dropped, and we found no substantive differences in the results. Please see Supplementary file 2, which contains the results of the analysed imputated data.

### Ethical consideration

Given that our study is based on the examination of a publicly available de-identified secondary dataset, there was no need for formal ethical approval to conduct this study. However, the World Health Organization (WHO) received ethical approval for the GSHS, and in Sierra Leone, institutional permission was sought from the Ministry of Health and Sanitation. Furthermore, both child consent and parental or guardian consent forms were obtained from adolescents below 18 years before their inclusion in the survey. Additionally, the WHO ensured that respondents who were 18 years old and above were provided informed consent, which was obtained using both written and verbal methods.

## Results

### Background characteristics of the school-going adolescents in Sierra Leone

Of the 1,467 school-going adolescents included in the study, 65.4% were aged 15 years and older, and 34.6% were aged 14 years and below. 52.2% of the school-going adolescents were females and 47.8% were males. More than half of the school-going adolescents were in Junior Secondary School (70.5%), were anxious (62.2%), had no suicidal ideation (88.7%), had not planned suicide (86.1%), and had not attempted suicide (85.3%). Most of the school-going adolescents did feel lonely (69.0%), had close friends (91.5%), experienced bullying (49.6%), were involved in sexual risk behaviour (70.7%), had peer support (81.6%), and were not truant at school (69.3%). Also, 81.4% of the school-going adolescents had parental support (Table 2).

**Table 2:** Background characteristics of the school-going adolescents in Sierra Leone (*n*=1467)

**Variables**	**Weighted frequency (*n*)**	**Weighted percentage (%)**
**Age (years)**		
≤14 years	512	34.6
≥15 years	955	65.4
**Grade**		
Junior secondary school (JSS)	935	70.5
Senior Secondary School (SSS)	532	29.5
**Gender**		
Female	685	52.2
Male	782	47.8
**Anxiety**		
No	571	37.8
Yes	896	62.2
**Suicidal ideation**		
No	1296	88.7
Yes	171	11.3
**Suicidal plan**		
No	1260	86.1
Yes	207	13.9
**Suicidal attempt**		
No	1243	85.3
Yes	224	14.7
**Felt lonely**		
No	460	31.0
Yes	1007	69.0
**Close friends**		
No	121	8.5
Yes	1346	91.5
**Alcohol use**		
No	1270	88.3
Yes	197	11.7
**Amphetamine use**		
No	1366	93.6
Yes	101	6.4
**Bullied**		
No	728	50.4
Yes	739	49.6
**Sexual risk behaviour**		
No	1029	70.7
Yes	438	29.3
**Peer support**		
No	289	18.4
Yes	1174	81.6
**School truancy**		
No	1002	69.3
Yes	465	30.7
**Parental support**		
No	282	18.6
Yes	1185	81.4

### Prevalence of cannabis use and its distribution across the explanatory variables

Table 3 presents the prevalence of cannabis use and its distribution across the explanatory variables. The prevalence of cannabis use was 5.1% [3.0, 8.6]. The prevalence of cannabis use was higher among school-going adolescents who were aged 15 years and above (6.6%), those in senior secondary school (10.3%), and male school-going adolescents (6.4%). Also, anxious school-going adolescents (6.7%), those who had suicidal ideations (10.2%), those who planned suicide (10.1%), those who attempted suicide (17.9%), those who felt lonely (6.1%), and those who had no close friends (5.7%). Cannabis use was prevalent among school-going adolescents who were bullied (8.2%), those who were involved in sexual risk behaviour (13.4%), those with no peer support (5.2%), and those who were truant at school (9.1%). Additionally, cannabis use prevalence was high among school-going adolescents who had no parental support (6.6%). Except for gender, feeling lonely, close friends, peer support, truancy in school and parental support, the remaining variables were statistically associated with cannabis use at *p*<0.05.

**Table 3:** Prevalence and distribution of cannabis use among school-going adolescents in Sierra Leone

**Variables**	**Cannabis use**	
	**No % [CI]**	**Yes % [CI]**
**Prevalence**	**94.9 [91.4,97.0]**	**5.1 [3.0, 8.6]**
**Age (years)**		
≤14 years	97.7 [94.4, 99.1]	2.3 [0.9, 5.6]
≥15 years	93.4 [88.4, 96.3]	6.6 [3.7, 11.6]
**Grade**		
Junior secondary school (JSS)	97.1 [95.1, 98.3]	2.9 [1.7, 4.9]
Senior Secondary School (SSS)	89.7 [80.3, 94.9]	10.3 [5.1, 19.7]
**Gender**		
Female	96.3 [91.0, 98.5]	3.7 [1.5, 9.0]
Male	93.6 [89.9, 96.0]	6.4 [4.0, 10.1]
**Anxiety**		
No	97.5 [95.4, 98.6]	2.5 [1.4, 4.6]
Yes	93.3 [87.1, 96.7]	6.7 [3.3, 12.9]
**Suicidal ideation**		
No	95.5 [91.8, 97.6]	4.5 [2.4, 8.2]
Yes	89.8 [80.8, 94.8]	10.2 [5.2, 19.2]
**Suicidal plan**		
No	95.7 [92.3, 97.6]	4.3 [2.4, 7.7]
Yes	89.9 [80.8, 94.9]	10.1 [5.1, 19.2]
**Suicidal attempt**		
No	97.1 [95.4, 98.2]	2.9 [1.8, 4.6]
Yes	76.7 [66.2, 84.7]	17.9 [8.4, 34.1]
**Felt lonely**		
No	97.0 [93.9, 98.6]	3.0 [1.4, 6.1]
Yes	93.9 [88.8, 96.8]	6.1 [3.2, 11.2]
**Close friends**		
No	94.3 [85.2, 97.9]	5.7 [2.1, 14.8]
Yes	94.9 [91.6, 97.0]	5.1 [3.0, 8.4]
**Alcohol use**		
No	96.9 [92.3, 98.8]	3.1 [1.2, 7.7]
Yes	79.9 [65.1, 89.4]	20.1 [10.6, 34.9]
**Amphetamine use**		
No	97.3 [93.3, 99.0]	2.7 [1.0, 6.7]
Yes	59.0 [42.7, 73.5]	41.0 [26.5, 57.3]
**Bullied**		
No	97.9 [96.1, 98.9]	2.1 [1.1, 3.9]
Yes	91.8 [85.5, 95.5]	8.2 [4.5, 14.5]
**Sexual risk behaviour**		
No	98.3 [97.6, 98.9]	1.7 [1.1, 2.4]
Yes	86.6 [78.0, 92.1]	13.4 [7.9, 22.0]
**Peer support**		
No	94.8 [89.5, 97.5]	5.2 [2.5, 10.5]
Yes	94.9 [90.6, 97.3]	5.1 [2.7, 9.4]
**School truancy**		
No	96.7 [90.4, 98.9]	3.3 [1.1, 9.6]
Yes	90.9 [85.3, 94.5]	9.1 [5.5, 14.7]
**Parental support**		
No	93.4 [88.7, 96.2]	6.6 [3.8, 11.3]
Yes	95.2 [90.4, 97.7]	4.8 [2.3, 9.6]

### Factors associated with cannabis use among the in-school adolescents in Sierra Leone

Table 4 shows the results of the factors associated with cannabis use among school-going adolescents in Sierra Leone. School-going adolescents who attempted suicide [aOR = 6.34, 95% CI = 1.71–23.45], used amphetamine [aOR = 15.84, 95% CI = 7.94–31.62] and were involved in sexual risk behaviour [aOR = 5.56, 95% CI = 2.18–14.20] were more likely to be associated with cannabis use.

**Table 4:** Factors associated with cannabis use among school-going adolescents in Sierra Leone

	**Cannabis use**	
**Variables**	**Model I** **cOR [95% CI]**	**Model II** **aOR [95% CI]**
**Age (years)**		
≤14 years	1.00	1.00
≥15 years	3.01* [0.99, 9.11]	1.20 [0.49, 2.93]
**Grade**		
Junior secondary school (JSS)	1.00	1.00
Senior Secondary School (SSS)	3.82*** [1.51, 9.64]	3.67 [0.86, 15.63]
**Gender**		
Female	1.00	-
Male	1.78 [0.76, 4.16]	-
**Anxiety**		
No	1.00	-
Yes	2.78 [0.95, 8.09]	-
**Suicidal ideation**		
No	1.00	1.00
Yes	2.43** [1.01, 5.84]	1.14 [0.57, 2.28]
**Suicidal plan**		
No	1.00	1.00
Yes	2.50** [1.23, 5.09]	0.96 [0.45, 2.05]
**Suicidal attempt**		
No	1.00	1.00
Yes	7.23*** [2.98, 17.54]	6.34* [1.71, 23.45]
**Felt lonely**		
No	1.00	-
Yes	2.12 [0.79, 5.70]	-
**Close friends**		
No	1.00	-
Yes	0.88 [0.39, 1.95]	-
**Alcohol use**		
No	1.00	1.00
Yes	7.78*** [1.87, 32.40]	1.44 [0.26, 7.82]
**Amphetamine use**		
No	1.00	1.00
Yes	25.23*** [8.79, 72.38]	15.84*** [7.94, 31.62]
**Bullied**		
No	1.00	1.00
Yes	4.19*** [1.67, 10.49]	1.90 [0.50, 7.13]
**Sexual risk behaviour**		
No	1.00	1.00
Yes	9.23*** [4.64, 18.37]	5.56*** [2.18, 14.20]
**Peer support**		
No	1.00	-
Yes	0.96 [0.36, 2.60]	-
**School truancy**		
No	1.00	-
Yes	2.87 [0.72, 11.50]	-
**Parental support**		
No	1.00	-
Yes	0.70 [0.24, 2.00]	-

*aOR* adjusted odds ratios, *cOR* crude odds ratios, *CI* Confidence Interval

* p< 0.05, ** p<; 0.01, *** p<; 0.001

## Discussion

Our study examined the prevalence of cannabis use and its associated factors among school-going adolescents in Sierra Leone. Our study found a 5.1% prevalence of cannabis use among school-going adolescents in Sierra Leone. Suicidal attempt, amphetamine use, and sexual risk behaviour were the factors associated with cannabis use among school-going adolescents in Sierra Leone.

The prevalence of 5.1% reported in this study is lower than the 5.3% reported in Ghana [4]. However, it is comparable to UNODC research [1], showing that teenage cannabis usage was low in Nigeria (4.4%) and Morocco (4.0%). In addition, recent research found that Namibia, Swaziland, and Mauritius had prevalence rates of prior 30-day cannabis usage of 5.3%, 4.6%, and 4.3%, respectively [12]. Still, the reported prevalence in the African region of 37.2% reported in Zambia [5], 26% reported in Port Harcourt in Nigeria [6], and 16.9% reported in KwaZulu-Natal in South Africa [10], is significantly higher than the current study's findings. Adolescents may be influenced by their friends or peers to try cannabis, either out of curiosity or to fit in [4]. Adolescents may use cannabis to cope with stress, trauma, or other psychosocial problems, such as poverty, violence, or family issues [15]. Adolescents may not be aware of the harmful effects of cannabis on their health, education, and prospects, or they may underestimate the risks [18].

Our study found an association between amphetamine use and a higher likelihood of cannabis use among school-going adolescents in Sierra Leone. The gateway theory suggests that using one substance, like amphetamines, might increase the likelihood of using other drugs, including cannabis [27]. Both amphetamines and cannabis can have mood-altering effects, and school-going adolescents struggling with depression, anxiety, or other mental health issues might use them to cope [28]. Adolescents who are more prone to risky behaviours in general might be more likely to experiment with multiple substances, including both amphetamines and cannabis [4].

The study found an association between cannabis use and sexual risk behaviours among school-going adolescents in Sierra Leone. School-going adolescents who engage in sexual risk behaviours might use cannabis to cope with emotional distress or adverse consequences arising from those behaviours [29]. Policymakers in Sierra Leone should provide comprehensive and age-appropriate sexual health education to promote safe sexual practices. They should also address underlying mental health issues that might contribute to both risk behaviours.

Our study found an association between attempted suicide and cannabis use among school-going adolescents in Sierra Leone. This finding is consistent with the previous studies [30, 31]. School-going adolescents struggling with suicidal thoughts or depression might use cannabis as a coping mechanism [32]. School-going adolescents who have experienced traumatic events might be more susceptible to both self-harm and substance misuse as coping mechanisms [33]. Government and policymakers in Sierra Leone should make accessible and culturally appropriate mental health services readily available for adolescents. They should Implement programs that raise awareness of suicide, reduce stigma, and provide support to individuals at risk. They should provide resources and support for school-going adolescents struggling with substance use.

### Policy and practice implication

The study's findings underscore the necessity of implementing preventive measures. Policies should be designed to promote the implementation of educational programmes in schools that specifically target school-going adolescents, aiming to raise awareness about the potential dangers associated with cannabis use, as well as other risky behaviours found in the study, such as suicidal attempts, amphetamine use, and sexual risk behaviour. The association between cannabis use and suicidal attempts indicates a necessity for enhanced mental health assistance in educational institutions and local communities. Policy initiatives should prioritise early intervention programmes aimed at identifying school-going adolescents who are at risk and facilitating their access to suitable services. It is imperative to establish community outreach initiatives aimed at educating parents and carers about the hazards associated with cannabis use and providing them with guidance on how to communicate with their children regarding this matter effectively. The study highlights a need for enhanced availability of healthcare services, namely mental health services, for school-going adolescents, and the reduction of stigma surrounding mental health problems to promote the willingness to seek care.

### Strengths and limitations of the study

This research has several limitations. First off, cannabis usage, the main outcome variable, was self-reported. Biases based on social desirability and systematic bias may affect self-report. Second, most measures used were single-item tests, which only permitted a limited variable evaluation. Thirdly, we cannot prove causation since the findings are based on a cross-sectional database. Finally, the research only included adolescents enrolled in school; 10 to 19-year-olds who were not enrolled were not included. As a result, not all adolescents in this age bracket are represented by the results. Despite these drawbacks, this is one of the first cross-sectional studies to examine the prevalence of cannabis use and its related characteristics among school-going adolescents using nationally representative data in Sierra Leone.

## Conclusion

A small but non-trivial minority of students use cannabis, and the cannabis users tend to report other concerning social issues and co-occurring drug use such as suicidal attempts, amphetamine use, and engaging in sexual risk behaviours. The study underscores the necessity of creating school-based intervention programmes due to the high occurrence and connection to other risky behaviours. These programmes should focus on preventing cannabis use and addressing the risk factors associated with it in school-going adolescents.

### Supplementary Information

**Supplementary Material 1.**

**Supplementary Material 2.**

## Data Availability

The dataset informing the findings of this study is publicly available. It can be freely accessed via the WHO NCD Microdata Repository https://extranet.who.int/ncdsmicrodata/index.php/catalog/GSHS.
