# Supplementary material for: Cannabis use and its psychosocial correlates among school-going adolescents in Sierra Leone
Source: BMC Public Health. 2024 Apr 8;24:986. doi: 10.1186/s12889-024-18491-0 (PMC11003035; doi:10.1186/s12889-024-18491-0)
Supplement: Supplementary file 2 — Supplementary Material 2. [file 12889_2024_18491_MOESM2_ESM.docx]

Table 3 presents the prevalence of cannabis use and its distribution across the explanatory variables. The prevalence of cannabis use was 5.5% [3.6, 8.1]. The prevalence of cannabis use was higher among school-going adolescents who were aged 15 years and above (79.1%), those in senior secondary school (55.2%), and male school-going adolescents (66.6%). Also, anxious school-going adolescents (81.8%), those who had suicidal ideations (30.4%), those who planned suicide (34.8%), and those who attempted suicide (58.0%). Cannabis use was prevalent among school-going adolescents who were bullied (85.3%), those who were involved in sexual risk behaviour (82.7%), and those who were truant at school (63.5%). Except for felt lonely, close friends, peer support, and parental support, the remaining variables were statistically associated with cannabis use at p<0.05.

**Table 3: Prevalence and distribution of cannabis use among school-going adolescents in Sierra Leone.**

| **Variables** | **Cannabis use** | |
| --- | --- | --- |
|  | **No % [CI]** | **Yes % [CI]** |
| **Prevalence** | **94.5 [91.9, 96.4]** | **5.5 [3.6, 8.1]** |
| **Age (years)** |  |  |
| ≤14 years | 36.5 [27.7, 46.3] | 20.9 [11.3, 35.4] |
| ≥15 years | 63.5 [53.7, 72.3] | 79.1 [64.6, 88.7] |
| **Grade** |  |  |
| Junior secondary school (JSS) | 72.3 [57.3, 83.5] | 44.8 [24.1, 67.6] |
| Senior Secondary School (SSS) | 27.7 [16.5, 42.7] | 55.2 [32.4, 75.9] |
| **Gender** |  |  |
| Female | 49.1 [41.9, 56.3] | 33.4 [23.4, 45.2] |
| Male | 50.9 [43.7, 58.1] | 66.6 [54.8, 76.6] |
| **Anxiety** |  |  |
| No | 36.1 [31.4, 41.1] | 18.2 [10.7, 29.4] |
| Yes | 63.9 [58.9, 68.6] | 81.8 [70.6, 89.3] |
| **Suicidal ideation** |  |  |
| No | 86.5 [81.6, 90.3] | 69.6 [55.5, 80.7] |
| Yes | 13.5 [9.7, 18.4] | 30.4 [19.3, 44.5] |
| **Suicidal plan** |  |  |
| No | 84.8 [79.6, 88.8] | 65.2 [53.3, 75.4] |
| Yes | 15.2 [11.2, 20.4] | 34.8 [24.6, 46.7] |
| **Suicidal attempt** |  |  |
| No | 83.1 [77.4, 87.6] | 42.0 [30.4, 54.6] |
| Yes | 16.9 [12.4, 22.6] | 58.0 [45.4, 69.6] |
| **Felt lonely** |  |  |
| No | 30.1 [26.6, 33.9] | 21.0 [13.7, 30.8] |
| Yes | 69.9 [66.1, 73.4] | 79.0 [69.2, 86.3] |
| **Close friends** |  |  |
| No | 9.2 [7.9, 10.7] | 10.8 [6.6, 17.2] |
| Yes | 90.8 [89.3, 92.1] | 89.2 [82.8, 93.4] |
| **Alcohol use** |  |  |
| No | 88.7 [84.9, 91.7] | 43.4 [26.3, 62.3] |
| Yes | 11.3 [8.3, 15.1] | 56.6 [37.7, 73.7] |
| **Amphetamine use** |  |  |
| No | 94.4 [92.7, 95.8] | 44.1 [29.7, 59.6] |
| Yes | 5.6 [42.7, 73.5] | 55.9 [40.4, 70.3] |
| **Bullied** |  |  |
| No | 44.8 [38.3, 51.6] | 14.7 [7.9, 25.5] |
| Yes | 55.2 [48.4, 61.7] | 85.3 [74.5, 92.1] |
| **Sexual risk behaviour** |  |  |
| No | 63.3 [57.5, 68.6] | 17.3 [11.0, 26.1] |
| Yes | 36.7 [31.4, 42.5] | 82.7 [73.9, 89.0] |
| **Peer support** |  |  |
| No | 19.6 [15.4, 24.7] | 18.9 [11.4, 29.6] |
| Yes | 80.4 [75.3, 84.6] | 81.1 [70.4, 88.6] |
| **School truancy** |  |  |
| No | 66.6 [61.1, 71.8] | 36.5 [20.5, 56.3] |
| Yes | 33.4 [28.2, 38.9] | 63.5 [43.7, 79.5] |
| **Parental support** |  |  |
| No | 17.5 [15.2, 20.2] | 19.2 [11.8, 29.7] |
| Yes | 82.5 [79.8, 84.8] | 80.8 [70.3, 88.2] |

Table 4 shows the results of the factors associated with cannabis use among school-going adolescents in Sierra Leone. School-going adolescents who attempted suicide [aOR = 3.01, 95% CI = 1.12–8.10], used amphetamine [aOR = 7.49, 95% CI = 5.39–10.40] and were involved in sexual risk behaviour [aOR = 3.24, 95% CI = 1.67–6.43] were more likely to be associated with cannabis use.

**Table 4: Factors associated with cannabis use among school-going adolescents in Sierra Leone.**

| **Variables** | **Cannabis use**  **aOR [95% CI]** |
| --- | --- |
| **Age (years)** |  |
| ≤14 years | 1.00 |
| ≥15 years | 1.18 [0.64, 2.17] |
| **Grade** |  |
| Junior secondary school (JSS) | 1.00 |
| Senior Secondary School (SSS) | 2.70 [1.01, 7.23] |
| **Gender** |  |
| Female | 1.00 |
| Male | 1.29 [0.75, 2.21] |
| **Anxiety** |  |
| No | 1.00 |
| Yes | 1.50 [0.60, 3.74] |
| **Suicidal ideation** |  |
| No | 1.00 |
| Yes | 1.00 [0.56, 1.77] |
| **Suicidal plan** |  |
| No | 1.00 |
| Yes | 1.27 [0.70, 2.29] |
| **Suicidal attempt** |  |
| No | 1.00 |
| Yes | 3.01*** [1.12, 8.10] |
| **Felt lonely** |  |
| No | 1.00 |
| Yes | 0.94 [0.53, 1.65] |
| **Close friends** |  |
| No | ­- |
| Yes | - |
| **Alcohol use** |  |
| No | 1.00 |
| Yes | 2.03 [0.65, 6.29] |
| **Amphetamine use** |  |
| No | 1.00 |
| Yes | 7.49*** [5.39, 10.40] |
| **Bullied** |  |
| No | 1.00 |
| Yes | 2.10 [0.74, 5.93] |
| **Sexual risk behaviour** |  |
| No | 1.00 |
| Yes | 3.24*** [1.67, 6.43] |
| **School truancy** |  |
| No | 1.00 |
| Yes | 1.34 [0.52, 3.42] |
| **Peer support** |  |
| No | - |
| Yes | - |
| **Parental support** |  |
| No | ­- |
| Yes | - |

aOR= adjusted odds ratios; CI Confidence Interval; * p< 0.05, ** p<; 0.01, *** p<; 0.001
